# Coexpression network analysis identified MT3 as a hub gene that promotes the chemoresistance of oral cancer by regulating the expression of YAP1

**DOI:** 10.1186/s12903-023-03600-z

**Published:** 2023-12-01

**Authors:** Jingyi Luo, Xin Liu, Yifei Zhang, Miao Yin, Li Xu, Menglei Cao, Bo Cheng, Sisi Yang

**Affiliations:** 1 Department of Stomatology, Zhongnan Hospital of Wuhan University, No. 169 Donghu Road, Wuchang District, Wuhan, 430071 China; 2 Department of Bacteriology and Immunology, Beijing Chest Hospital, Capital Medical University/Beijing Tuberculosis and Thoracic Tumor Research Institute, Beijing, China; 3 Department of Stomatology, Tongji Medical College, The Central Hospital of Wuhan, Huazhong University of Science and Technology, Wuhan, China

**Keywords:** Oral cancer, Drug resistance, MT3, YAP1, WGCNA

## Abstract

**Background:**

Oral cancer is considered one of the most malignant types of tumors and is known for its high likelihood of recurrence and metastasis. During clinical treatment, patients with oral cancer often develop resistance to chemotherapy, making the treatment process challenging. The purpose of this study was to investigate the genes related to chemotherapy resistance and their mechanisms in oral cancer patients.

**Methods:**

The “limma” package was used to identify the differentially expressed genes between tumor and normal tissues from TCGA dataset. Subsequently, the “WGCNA” package was utilized to discover genes associated with chemoresistance. Cisplatin-resistant oral cancer cell lines were obtained through exposure to gradually increasing doses of cisplatin. SiRNA was used to knock down the MT3 and YAP1 genes to validate their functions. Finally, the therapeutic efficacy of combining a YAP1 inhibitor with cisplatin was confirmed by inoculating an oral cancer cell line in mice.

**Results:**

In our study, we analyzed 43 OSCC samples and identified 724 different genes using the weighted gene coexpression network analysis (WGCNA) method. Among these genes, MT3 stood out as strongly associated with chemotherapy resistance. Patients with high MT3 expression had worse prognoses, and MT3 levels were elevated in drug-resistant patients. Knocking down MT3 reversed tumor cell chemoresistance. We also observed that MT3 increased the expression of YAP1, potentially contributing to chemotherapy resistance by inducing tumor stemness through YAP1. In animal models, using YAP1 inhibitors improved the effectiveness of cisplatin in treating chemoresistant oral cancer.

**Conclusions:**

MT3 is related to chemotherapy resistance, which may be caused by its promotion of YAP1 expression and induction of tumor cell stemness. Inhibiting the activity of MT3 and YAP1 is helpful for increasing chemotherapy sensitivity.

**Supplementary Information:**

The online version contains supplementary material available at 10.1186/s12903-023-03600-z.

## Introduction

Oral cancer has become a global health problem, with more than 355,000 new cases reported annually [1]. Nearly 90% of oropharyngeal cancers are oral squamous cell carcinomas (OSCC) [2], which are highly malignant tumors that lead to death in half of patients due to local recurrence and distant metastasis. Despite advancements in the diagnosis and treatment of oral cancer, its prognosis remains poor, with a five-year survival rate of less than 50% [3]. Oral cancer not only affects a patient’s appearance but also can invade the opening and closing muscles and temporomandibular joint, resulting in difficulty with chewing and swallowing [4, 5]. Surgical resection is the first line of treatment for oral cancer, but many patients are diagnosed in the advanced stages of the tumor [6]. When surgery is not an option, radiotherapy, chemotherapy, or immunotherapy is often used. Cisplatin and 5-fluorouracil (5-FU) are the most commonly used chemotherapy drugs for OSCC patients [7], but drug resistance often develops over time during treatment, leading to treatment failure [8].

In recent years, various mechanisms of chemoresistance in oral cancer have been discovered, including DNA damage and repair, transport processes, programmed cell death, and the tumor microenvironment (TME) [9]. Our study assessed a cohort of oral cancer patients who were treated with chemotherapy to investigate gene expression differences between chemotherapy-resistant (CR) and chemotherapy-sensitive (CS) patients. Through weighted gene coexpression network analysis (WGCNA) [10], we found that MT3 is associated with cisplatin resistance in oral cancer, and our results suggest a different mechanism of chemotherapy resistance in oral cancer than previously known. MT3 regulates drug resistance in tumor cells by regulating the expression of YAP1, a protein that has been linked to tumor proliferation, invasion, and migration [11, 12].

Chemotherapy resistance in oral cancer creates significant challenges for patient treatment and results in a poor prognosis for oral cancer. This study aimed to identify the genes most closely linked to drug resistance in oral cancer. Using WGCNA, we identified gene modules that are closely related to drug resistance and discovered that the MT3-YAP1 axis plays a critical role in chemoresistance in oral cancer.

## Methods

### Data collection and processing

The gene expression data of oral cancer patients were downloaded from the TCGA OSCC. We selected patients with cisplatin treatment and clear outcomes. Then, we filtered and standardized the data. Ultimately, a total of 72 patients with detailed clinical information and 724 genes were used for the subsequent analysis. All data were analyzed by R.4.0.2.

### Identification of differentially expressed genes and weighted coexpression network construction

We used the “limma” package to identify the differentially expressed genes between CR patients and CS patients. The genes with |logFoldChange| > 0 were considered significantly differentially expressed genes. We used “WGCNA” for the gene coexpression network construction, and the soft threshold for network construction was selected as 5. Then, we defined gene modules by gene branches using a dynamic tree-cutting algorithm. We displayed the relationship between gene modules and clinical signatures by a heatmap.

### Survival analysis and functional enrichment analysis

Patients were divided into two groups according to the expression level of MT3. The R packages “survival” and “ggplot” were applied to perform the survival analysis. We identified the differentially expressed genes between the high and low MT3 expression groups by using the “limma” package. Then, the functional enrichment analysis was performed via the “clusterProfiler” package.

### Human samples, animals and cell lines

Paraffin-embedded tumor tissues were obtained from oral cancer patients at Zhongnan Hospital of Wuhan University. Ethical permission was granted by the Medical Ethics Committee, Zhongnan Hospital of Wuhan University. Six-week-old female NSG mice were purchased from HFK Inc. (Beijing, China) and raised in an SPF grade environment. In all animal experiments, we complied with the ethical norms of animal experiments. CAL27 and Fadu oral cancer cell lines were obtained from ATCC and cultured in DMEM. By continuously exposing CAL27 and Fadu oral cancer cell lines to stepwise increasing concentrations of cisplatin over 6 months, CIS-resistant CAL27-CISR and Fadu-CISR cells were generated. All media were supplemented with 10% fetal bovine serum (FBS) (Gibco, USA) and 2 mM L-glutamine (Gibco, USA). All cells were grown at 37 °C in a 5% CO2 incubator.

### Histological and immunohistochemical staining

Tumor tissues were collected from patients with oral cancer at Zhongnan Hospital. Then, the samples were fixed in 10% formalin, embedded in paraffin and sectioned for H&E staining. The slides of tumor tissues were incubated with anti-MT3 or anti-Yap at 4 °C for 12 h. Afterward, the slides were incubated with HRP-conjugated secondary antibodies for 1 h at room temperature, followed by development with DAB substrate and counterstaining with hematoxylin.

### Knockdown of MT3 and YAP1

Generation of MT3 or YAP1 knockdown using CRISPR‒Cas9. Fadu and CAL27 oral cancer cell lines were transfected with sgRNA against MT3 or YAP1 cloned in PX459 (Addgene, USA). Briefly, 2 × 10^5^ Fadu and CAL27 oral cancer cells were seeded in 6-well plates for 24 h. Fadu and CAL27 cells were transfected with 5 µg of PX459 using Lipofectamine 8000 (Beyotime, China) in Gibco Opti-MEM reduced serum medium (Thermo Fisher Scientific, USA). After 48 h, puromycin (2 µg/ml) was used for selection for 14 days, and cells were maintained in puromycin (0.5 µg/ml).

The following primers were used:

- SGGFP: 5’-CACCGGGGCGAGGAGCTGTTCACCG-3’;
- MT3-SG1: 5’- GTGGCGTCGCCCTCTCTAGGTGG-3’;
- MT3-SG2: 5’- GCGGACTCCTGCAAGTGCGAGGG-3’;
- YAP1-SG1: 5’- GATGATGTACCTCTGCCAGC-3’; and
- YAP1-SG2: 5’- GGGACAGCATGGCCTTCCTG-3’.

### Western blotting

Cells were collected and lysed in RIPA lysis buffer at 4 °C for 30 min. Then, we centrifuged the pyrolysis solution at 12,000 × g for 15 min and collected the supernatant. After the determination of concentration, the protein was run on an SDS‒PAGE gel and transferred to a nitrocellulose membrane. The antibodies we used included anti-actin, anti-MT3, and anti-YAP1. The original gels and multiple exposure images are shown in Additional file 2.

### Real-time PCR

Total RNA was extracted by using TRIzol (Beyotime, Shanghai) and reverse transcribed into cDNA by using a TAKARA reverse cDNA kit (TAKARA, Japan).

### CCK-8 assay

Cell viability was assessed using the CCK-8 assay (Solarbio, China). Fadu and CAL27 oral cancer cells were seeded into 96-well plates in 100 µL medium and incubated for 48 h. The absorbance was measured by spectrophotometry following the manufacturer’s protocol.

### Animal experiments and treatment protocol

Nude mice were purchased from HFK Animal BIOSCIENCE Co., LTD (Beijing) and kept in compliance with the Guidelines of Experimental Animal Welfare Ethics Committee, Zhongnan Hospital of Wuhan University. For the xenograft tumor experiment, the mice were randomly divided into 4 groups (6 mice/group) and subcutaneously inoculated with 1 × 10^6^ Fadu-CISR or CAL27-CISR cells and intraperitoneally injected with DMSO (control group, 100 µl), verteporfin (100 µl), CIS (5 mg/kg) or CIS (5 mg/kg) combined with verteporfin (100 µl). The length and width of transplanted tumors were measured every 5 days, tumor volume was calculated as (length × width^2^) π/6, and a survival curve was drawn.

## Results

### Identification of differentially expressed genes in the TCGA

The gene expression matrix was obtained from the TCGA, which contains information on 23 CR patients and 10 CS patients. We set a loose threshold (p < 0.05 and |logFoldChange| > 0) to obtain the differentially expressed genes (DEGs). In summary, there were 291 upregulated genes and 431 downregulated genes between chemotherapy-resistant and chemotherapy-sensitive patients.

### Weighted Co-expression Network Construction and Identification of Hub Genes

**Fig. 1:**
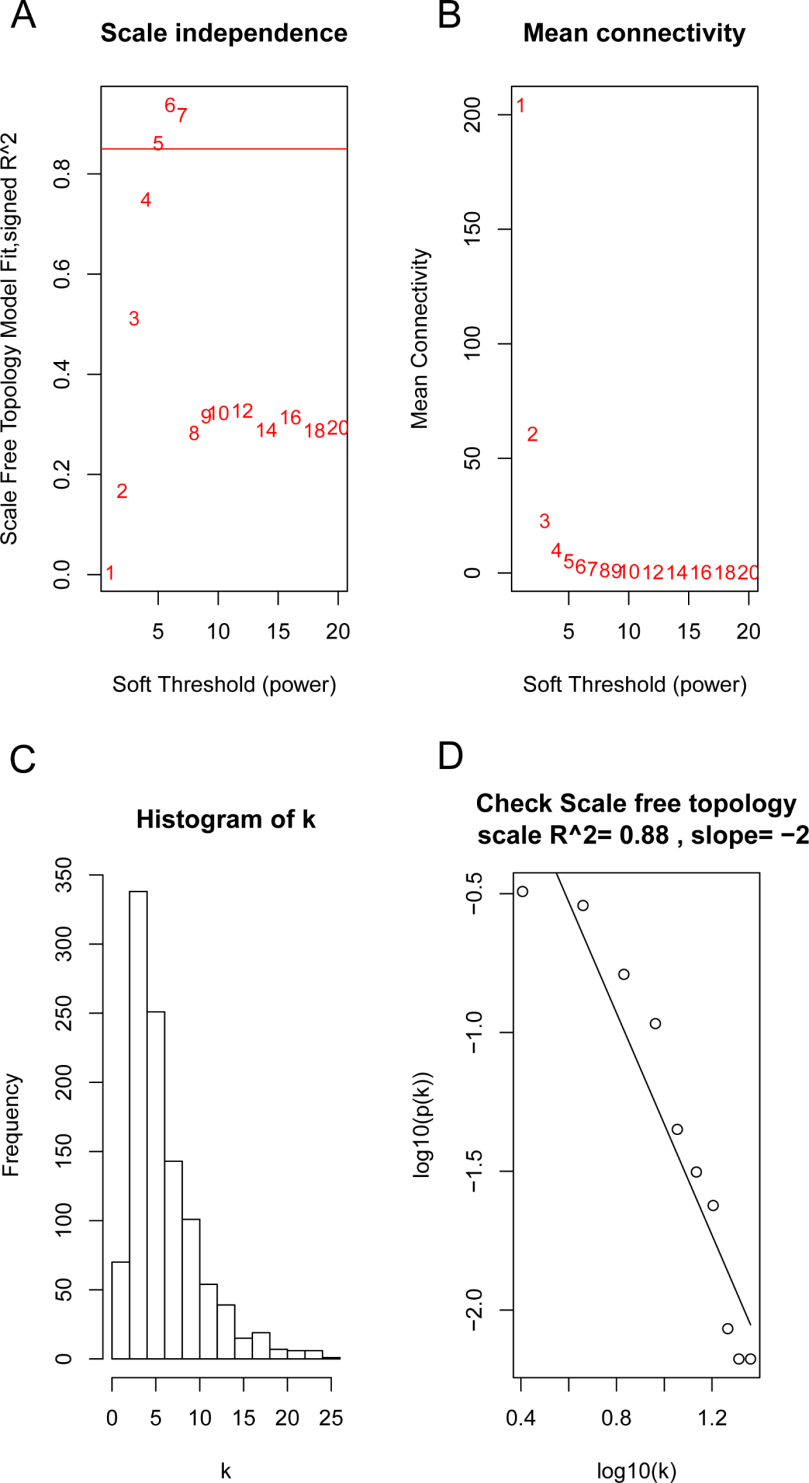
Calculation of soft threhold power in the WGCNA. (**A**) The scale-free fit index was analyzed for various soft‐thresholding powers (β). (**B**) Analysis of the mean connectivity for various soft‐thresholding powers. (**C**) Connectivity distribution (β = 5). (**D**) Analysis of the scale-free topology (β = 5)

**Fig. 2:**
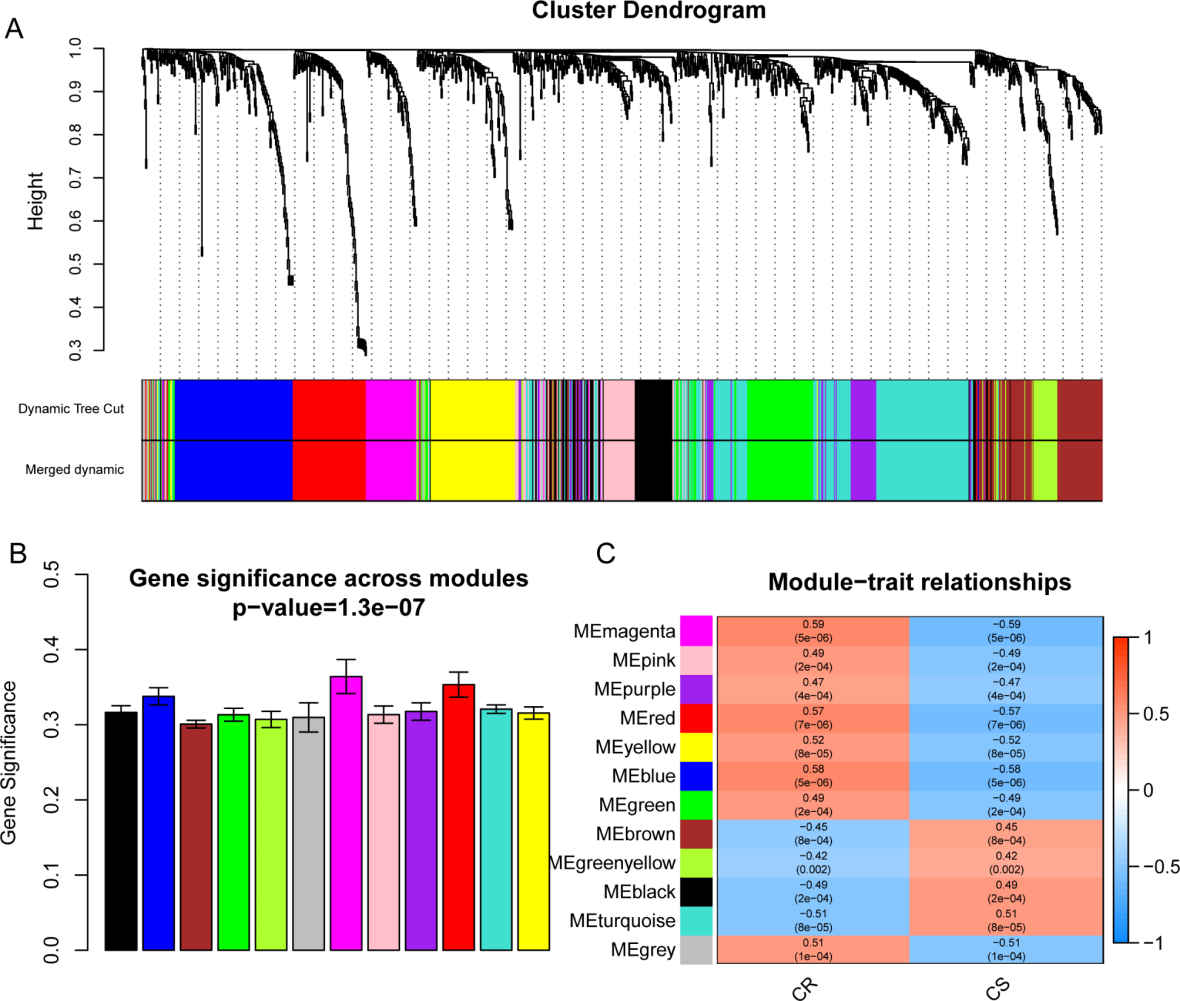
Identification of modules associated with chemoresistance in oral cancer. (**A**) Dendrogram of all differentially expressed genes clustered based on a dissimilarity measure (1-TOM). (**B**) Distribution of average gene significance and errors in the modules related to chemoresistance in oral cancer. (**C**) Heatmap of the correlation between module eigengenes and chemoresistance phenotype. TOM, topological overlap matrix

The R package “WGCNA” was applied to identify the gene modules in DEGs. According to the scale-free topology model, we selected β = 5 (R^2^ = 0.88) to establish the gene coexpression network (Fig. 1A-D). The gene modules were derived from the topological overlap matrix (TOM), and the minimum module size was set at 30. We set the threshold at 4 to merge the gene modules in which eleven gene modules were constructed (Fig. 2A-B). To find the module most relevant to chemotherapy resistance, we correlated the gene modules to clinical characteristics. The results showed that genes in the magenta modules were most significantly correlated with chemotherapy resistance (Fig. 2C).

### MT3 is the hub gene related to chemotherapy resistance

**Fig. 3:**
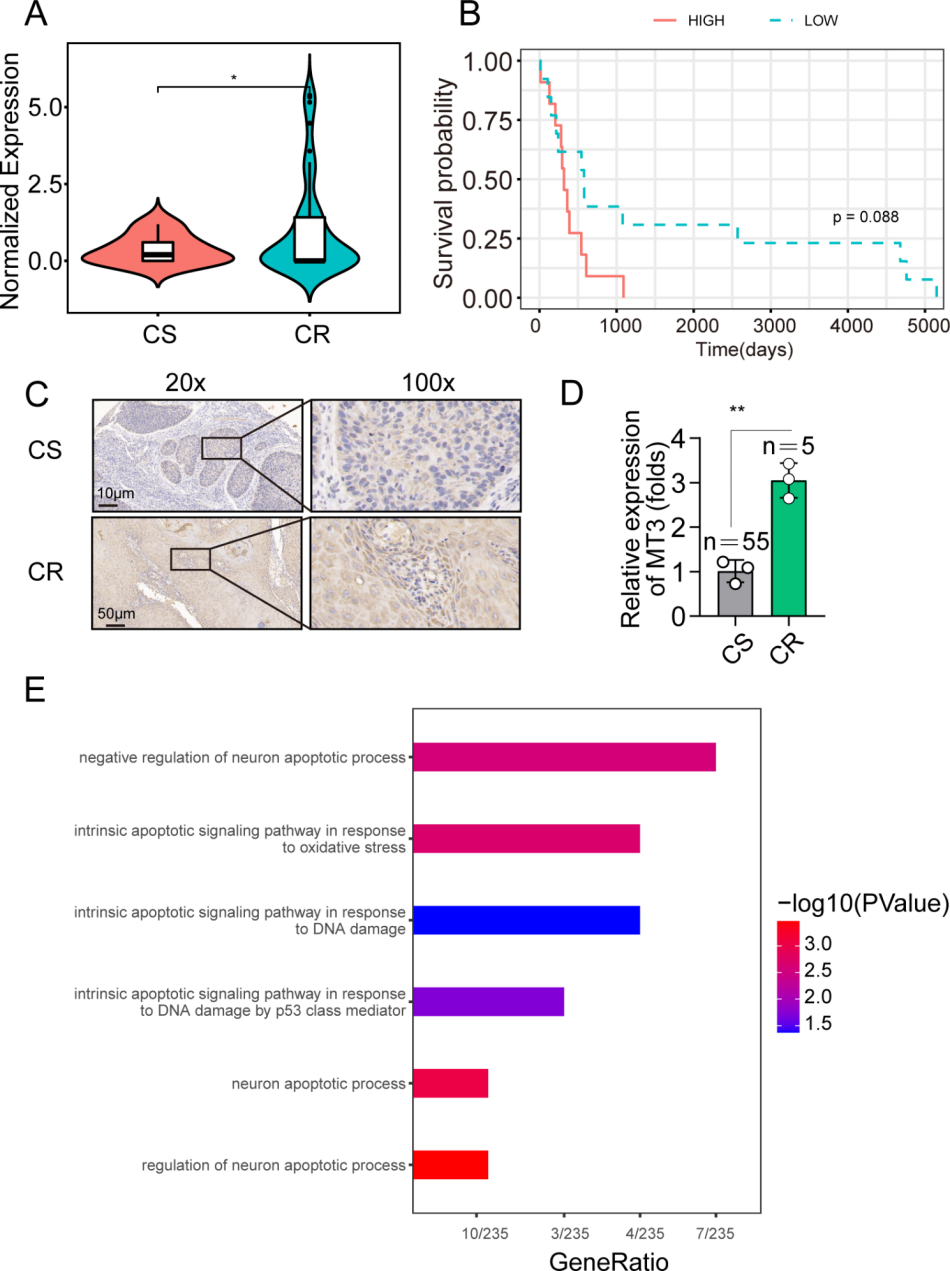
MT3 is highly expressed in chemotherapy-resistant (CR) OSCC tissue. (**A**) Transcription level of MT3 in chemotherapy-resistant (CR) and chemotherapy-sensitive (CS) patients in the cohort. (**B**) Overall survival analysis of patients with high/low MT3 expression. (**C**) Representative IHC images of MT3 expression in CR and CS tissues. (**D**) Comparison of MT3 IHC staining intensity in CR and CS tissues. (**E**) KEGG pathway analysis of modules in the chemoresistance phenotype

We applied the threshold of weighted and standard Pearson’s correlation coefficient > 0.85 (cor.weighted > |0.50| and cor.standard > |0.40|). Table 1 shows the top 5 genes in the module of interest by weighted correlation. MT3 was the top-ranked gene; it is a member of the metallothionein family, which participates in the regulation of metal homeostasis and has a high affinity for heavy metals. MT3 had the strongest relationship with chemotherapy resistance. Then, we compared the transcription level of MT3 in CR and CS patients in the cohort and found that MT3 expression was significantly higher in CR patients (Fig. 3A). Figure 3B shows the overall survival of patients with high or low MT3 expression, although there was no significant difference, the reason may be the limited number of samples. Furthermore, to validate our findings in clinical patients, we performed immunohistochemistry (IHC) staining on tissue samples from patients who received chemotherapy treatment and showed a response or no response. The CR patients had higher MT3 staining levels than CS patients. The IHC results proved that MT3 was highly expressed in CR patients (Fig. 3C-D). To investigate the potential biological differences, we compared the DEGs between patients with high and low MT3 expression by using the “limma” package. Then, we performed KEGG pathway analysis (Fig. 3E). Interestingly, pathways related to the apoptotic process, DNA damage, and oxidative stress were enriched, and all these pathways are associated with the killing effect of chemotherapy on cancer cells.

**Table 1:** The hub genes in the magenta module ranked by weighted correlation

Gene Name	cor.Weighted	cor.Standard	logFC	*P* value
MT3	0.83786585	0.438161556	0.744148469	0.009834297
HOXA9	0.788693398	0.489490152	-1.895209694	0.018509561
RXFP4	0.778212331	0.499998173	0.896116837	0.016866029
WFDC10B	0.758901882	0.45923419	0.666102551	0.022549376
C11orf86	0.714123707	0.455524596	-2.249766837	0.008307482

Abbreviations: FC, fold change

### Validation of the function of MT3 in cisplatin-resistant cell lines

**Fig. 4:**
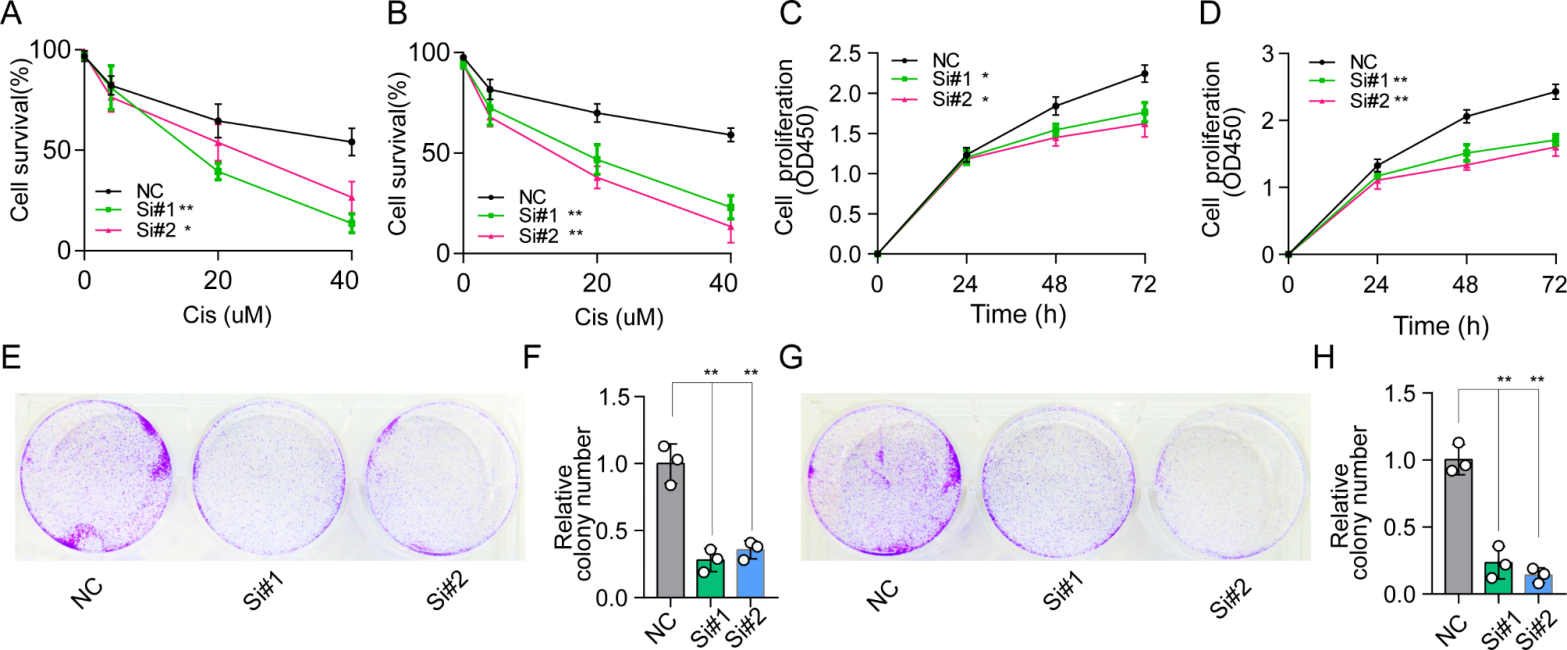
MT3 is crucial for the CIS resistance of OSCC cells. (**A-B**) Cell survival declined after MT3 knockdown in CAL27-CISR (CIS-resistant CAL27 cell line) or Fadu-CISR cells (CIS-resistant Fadu cell line). (**C-D**) Cell proliferation was suppressed after MT3 knockdown in CAL27-CISR or Fadu-CISR cells for 24 h, 48 and 72 h. (**E, G**) Representative images of colony formation. (**F, H**) Compared with the NC group, the number of colonies was reduced after MT3 knockdown in CAL27-CISR or Fadu-CISR cells. All results are presented as the mean ± SEM of three independent experiments. (p < 0.05*, p < 0.01**, p < 0.001***)

Herein, CAL27 and Fadu oral cancer cell lines were continuously exposed to stepwise increasing concentrations of cisplatin over 6 months. As anticipated, persistent CIS treatment led to the successful generation of CIS-resistant CAL27-CISR and Fadu-CISR cells. Knockdown of MT3 attenuated the CIS resistance of CAL27-CISR and Fadu-CISR cells (Fig. 4A-B). Furthermore, knockdown of MT3 disrupted the growth and colony formation of CISR cells (Fig. 4C-H). These results suggested that MT3 plays a crucial role in the development of CIS resistance.

### MT3 promotes chemotherapy resistance by regulating the expression of YAP1

**Fig. 5:**
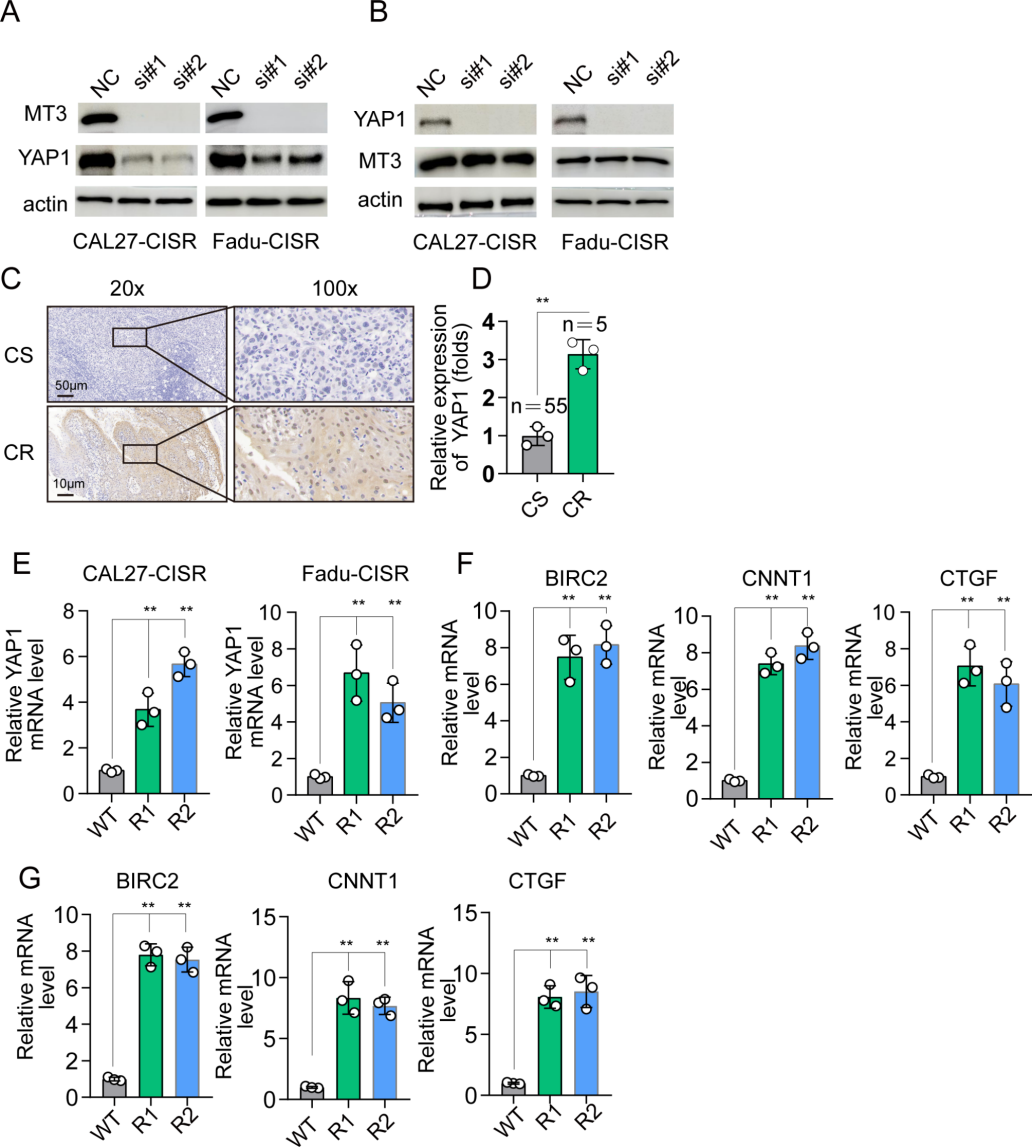
MT3 promotes cisplatin resistance by modulating YAP activation in OSCC. (**A-B**) Western blotting showed that YAP1 expression was decreased after MT3 knockdown, while the expression of MT3 was not changed after YAP1 knockdown in CAL27-CISR or Fadu-CISR cells. (**C-D**) Immunohistochemical staining of YAP1 in chemotherapy-resistant OSCC tissue (CR) and chemotherapy-sensitive tissue (CS). p < 0.01**. (**E**) qRT‒PCR results showing YAP1 mRNA levels in CAL27-CISR or Fadu-CISR cells. (**F**) qRT‒PCR results showing downstream target genes of YAP1, BIRC2, CNNT1, and CTGF mRNA levels in CAL27-CISR cells. (**G**) BIRC2, CNNT1, and CTGF mRNA levels in Fadu-CISR cells. n = 3, p < 0.01**

Next, we explored how MT3 promotes chemotherapy resistance in OSCC cells. Previous studies have reported that inhibition of YAP/TAZ not only disrupts tumorigenesis and tumor progression but also has the potential to sensitize tumor cells to chemotherapy or targeted therapies. Hence, we decided to investigate whether MT3 promotes cisplatin resistance by modulating YAP activation in OSCC. Western blot analysis showed that MT3 knockdown significantly decreased the expression of YAP1 (Fig. 5A). However, YAP1 knockdown had no effect on MT3 expression (Fig. 5B). Next, we detected the expression of YAP1 between the CR and the CS. High expression of YAP1 was verified by IHC staining in CIS-resistant OSCC tissue (Fig. 5C-D). Furthermore, increased levels of YAP1 and its downstream target genes were observed in CAL27-CISR and Fadu-CISR cells (Fig. 5E-G). To further investigate the impact of YAP1 on chemoresistance, YAP1 was knocked down in CAL27-CISR or Fadu-CISR cells (Fig. 5B). The results showed that knockdown of YAP1 not only reduced cancer cell survival (Additional file 1. Figure S1A) and cell growth (Additional file 1. Figure S1B) but also suppressed colony formation (Additional file 1. Figure S1C-D) in CAL27-CISR or Fadu-CISR cells. Collectively, these results revealed that MT3 promotes the chemoresistance of CAL27-CISR and Fadu-CISR cells by enhancing the expression of YAP1.

### YAP1 inhibition enhances the therapeutic effect of cisplatin

**Fig. 6:**
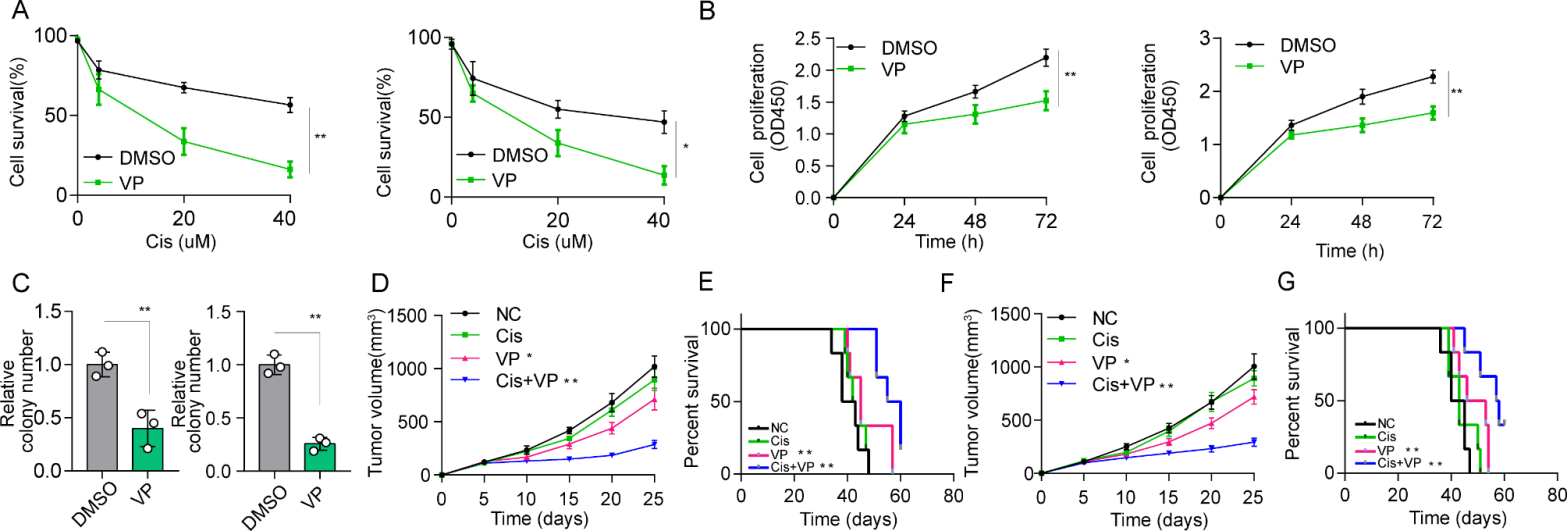
YAP1 inhibition suppresses the development of CIS resistance. (**A**) Cell survival declined after verteporfin (VP) treatment in CAL27-CISR or Fadu-CISR cells. (**B**) Cell proliferation was suppressed after VP treatment in CAL27-CISR or Fadu-CISR cells for 24 h, 48 and 72 h. (**C**) Compared with the DMSO group, the number of colonies was reduced after VP treatment in CAL27-CISR or Fadu-CISR cells. The results are presented as the mean ± SEM of three independent experiments. (**D-G**) CAL27-CISR or Fadu-CISR cells were subcutaneously inoculated into nude mice (n = 6) and then treated with DMSO (negative control), cisplatin, verteporfin, or cisplatin combined with verteporfin. Tumor growth (D, F) and survival analysis (E, G) were determined as indicated. (p < 0.05*, p < 0.01**, p < 0.001***)

Considering the key role of the MT3-YAP1 axis in regulating OSCC chemotherapy resistance, it might be feasible to retard tumor growth by suppressing YAP1. Herein, we used verteporfin, a small molecule inhibitor of YAP1, in combination with cisplatin-based chemotherapy to treat tumor-bearing mice. Treatment with verteporfin significantly strengthened the cytotoxicity of CIS to CAL27-CISR and Fadu-CISR cells (Fig. 6A). More importantly, verteporfin also suppressed growth (Fig. 6B) and colony formation (Fig. 6C) in CAL27-CISR and Fadu-CISR cells.

Since blockade of YAP1 in vitro plays a vital role in impairing the occurrence of CIS resistance, blocking YAP1 functions in vivo may have therapeutic benefits for cisplatin treatment-resistant tumors. We generated a xenograft mouse model using CAL27-CISR or Fadu-CISR cells injected subcutaneously and then treated the mice with DMSO, verteporfin, CIS or CIS combined with verteporfin. Compared to VP or CIS alone, the combination of verteporfin and CIS notably inhibited tumor growth and prolonged the survival time of mice bearing CAL27-CISR cell-derived tumors (Fig. 6D and E). The same results were observed in mice bearing Fadu-CISR cell-derived tumors (Fig. 6F and G). These results suggested that the application of the YAP1 inhibitor VP could strengthen the anticancer effects of traditional chemotherapeutics, providing a novel strategy for clinical OSCC treatment.

## Discussion

Oral cancer is the most serious oral health problem, and the number of new oral cancer patients is increasing every year. Smoking, chewing betelnut, excessive drinking and other bad habits can cause oral cancer [13]; the incidence of oral cancer remains high especially in South Asia and other regions where chewing betelnut is common. Similarly, the age of oral cancer patients is also decreasing [14], which may be related to young people’s open sexual behavior since HPV is also one of the causes of oral cancer [15]. Most patients present with oral cancer that has already metastasized at diagnosis and can only be treated with chemotherapy. Reversing the drug resistance of oral cancer cells is of great significance for improving therapeutic efficacy.

To investigate the potential mechanism of drug resistance in oral cancer, we used bioinformatics methods to analyze the TCGA dataset of oral cancer patients who received chemotherapy. According to the WGCNA results, MT3 is most closely related to drug resistance in patients with oral cancer, and patients with high expression of MT3 have a worse prognosis. MT3 is a member of the mammalian metallothionein (MT) family and is mainly expressed in the central nervous system (CNS) [16]. Some studies have shown that MT3 is highly expressed in malignant astrocytoma cells [17]. However, there has been no report on MT3 and chemoresistance in oral cancer. We found that MT3 expression was higher in chemotherapy-resistant oral cancer patients than in chemotherapy-sensitive patients. We performed differential gene expression analysis of samples from tumor patients with high and low expression of MT3, we subsequent analysis revealed that the differentially expressed genes were enriched in the response to oxidative stress and DNA damage-related pathways. We obtained cisplatin-resistant CAL27 and Fadu cell lines by gradually increasing the concentration of cisplatin in the medium. After knockdown of MT3 in the cisplatin-resistant cell line, the cells showed higher sensitivity to cisplatin. We found that MT3 may mediate the chemotherapy resistance of oral cancer through YAP1. MT3 expression is significantly correlated with YAP1 expression. The expression of YAP1 in drug-resistant cells is also significantly reduced after knockdown of MT3, but knockdown of YAP1 does not affect the expression of MT3, which indicates that MT3 is the upstream protein regulating YAP1. It has been reported that YAP1 promotes the stemness and drug resistance of many tumors, and here, we proved that it is highly expressed in chemoresistant oral cancer tissues. Finally, we verified this result in an animal model. We treated mice inoculated with oral cancer and found that a YAP1 inhibitor can effectively suppress the cisplatin resistance of OSCC tumor cells.

In the present study, we propose a new drug resistance pathway for oral cancer cells; that is, oral cancer cells regulate cell chemoresistance through the MT3-YAP1 axis. The expression of MT3 in oral cancer cells promotes the expression of YAP1, thereby regulating the stemness of tumor cells. However, how MT3 is regulated in chemoresistant cells is still unclear, and the mechanism of its regulation of YAP1 expression also needs to be studied in detail. Nevertheless, this study has proven that the use of YAP1 inhibitors can effectively reverse the chemoresistance of oral cancer, providing suggestions for the current clinical treatment of oral cancer.

### Electronic supplementary material

Below is the link to the electronic supplementary material.

Supplementary Material 1

Supplementary Material 2

## Data Availability

The datasets presented in this study can be found in GEO (https://www.ncbi.nlm.nih.gov/) and TCGA (https://www.genome.gov/Funded-Programs-Projects/Cancer-Genome-Atlas) databases.

## List of abbreviations

WGCNA: weighted gene coexpression network analysis
OSCC: oral squamous cell carcinomas
5-FU: 5-fluorouracil
TME: tumor microenvironment
CR: chemotherapy-resistant
CS: chemotherapy-sensitive
TOM: topological overlap matrix
IHC: immunohistochemistry
MT: mammalian metallothionein
CNS: central nervous system
