# Supplementary material for: Coexpression network analysis identified MT3 as a hub gene that promotes the chemoresistance of oral cancer by regulating the expression of YAP1
Source: BMC Oral Health. 2023 Dec 1;23:954. doi: 10.1186/s12903-023-03600-z (PMC10693099; doi:10.1186/s12903-023-03600-z)
Supplement: Supplementary file 1 — Supplementary Material 1 [file 12903_2023_3600_MOESM1_ESM.docx]

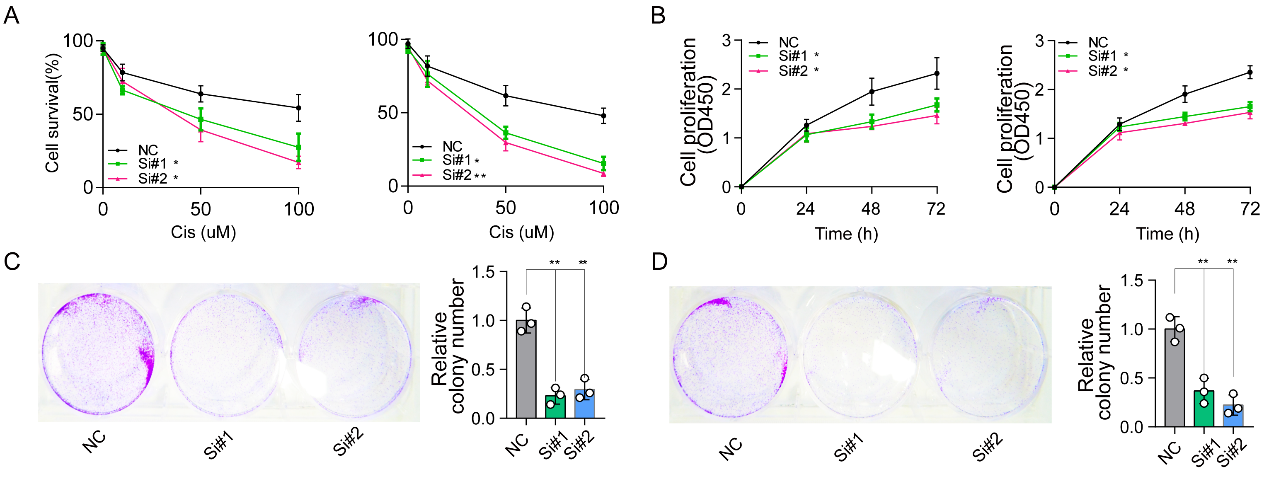


Figure S1 YAP1 promotes the development of CIS resistance. (A) Cell survival under exposure to cisplatin was declined after YAP1-knockdown in CAL27-CISR or Fadu-CISR cells. (B) Cell proliferation was suppressed when YAP1-knockdown in CAL27-CISR or Fadu-CISR cells after 24h, 48h and 72h. (C, D) Colony formation of CAL27-CISR or Fadu-CISR cells with or without YAP1-knockdown. The results are presented as the mean ± SEM of three independent experiments. (p < 0.05*, p < 0.01**, p < 0.001***).
