# Supplementary material for: Coexpression network analysis identified MT3 as a hub gene that promotes the chemoresistance of oral cancer by regulating the expression of YAP1
Source: BMC Oral Health. 2023 Dec 1;23:954. doi: 10.1186/s12903-023-03600-z (PMC10693099; doi:10.1186/s12903-023-03600-z)
Supplement: Supplementary file 2 — Supplementary Material 2 [file 12903_2023_3600_MOESM2_ESM.pdf]

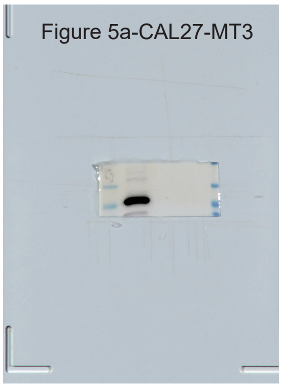
Figure 5a-CAL27-MT3

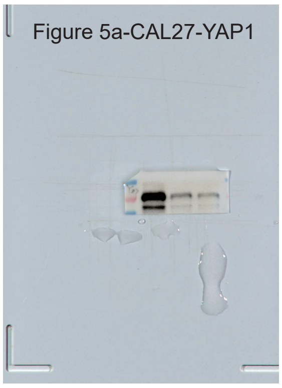
Figure 5a-CAL27-YAP1

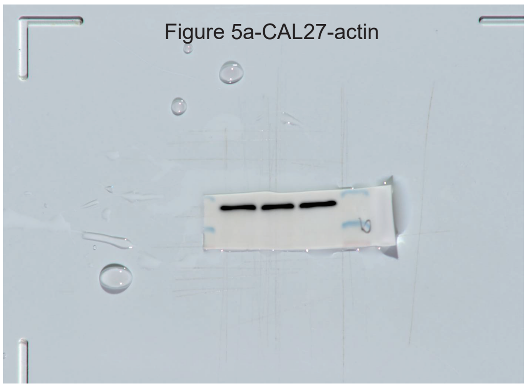
Figure 5a-CAL27-actin

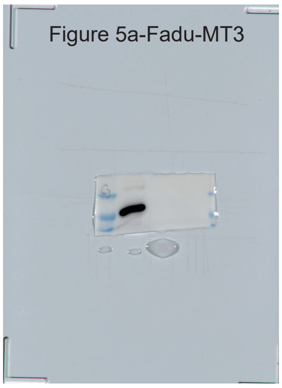
Figure 5a-Fadu-MT3

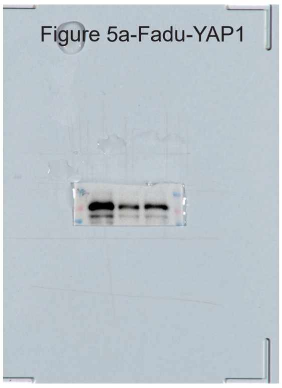
Figure 5a-Fadu-YAP1

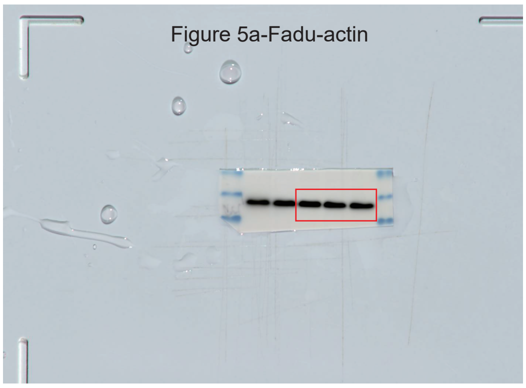
Figure 5a-Fadu-actin

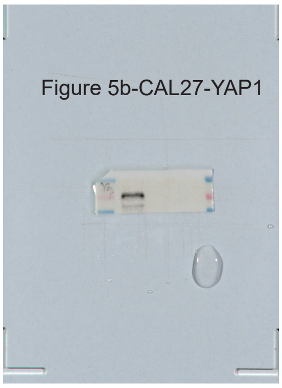
Figure 5b-CAL27-YAP1

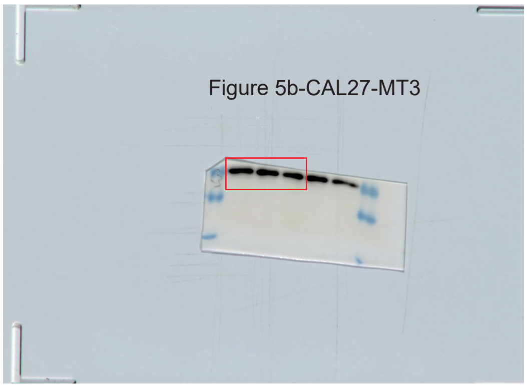
Figure 5b-CAL27-MT3

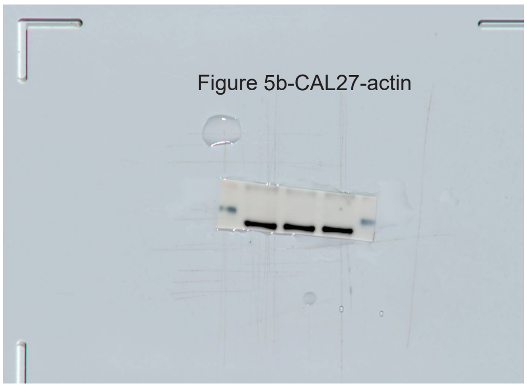
Figure 5b-CAL27-actin

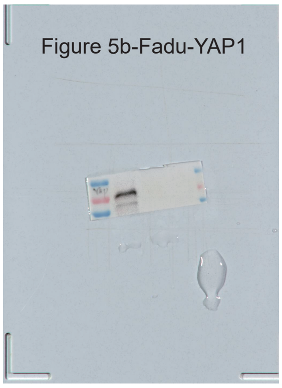
Figure 5b-Fadu-YAP1

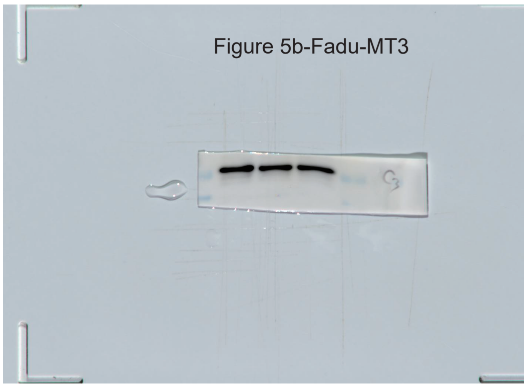
Figure 5b-Fadu-MT3

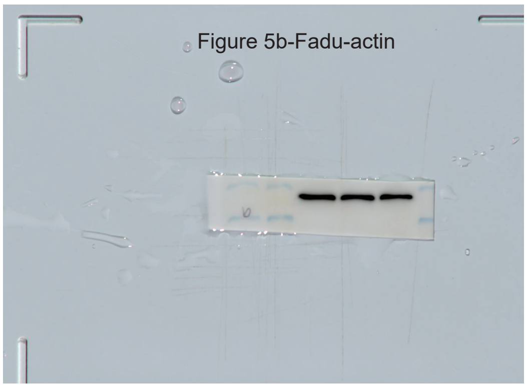
Figure 5b-Fadu-actin
